# “More than Hunger”: Experiences of Food Insecurity Among South Asian International Graduate Students at a U.S. University

**DOI:** 10.3390/nu17152508

**Published:** 2025-07-30

**Authors:** Lisa Henry, Doug Henry, Eva Perez Zepeda

**Affiliations:** 1 ImpactLab for Applied Social Science Research, University of North Texas, Denton, TX 76203, USA; doug.henry@unt.edu; 2 University of North Texas Health Science Center, ImpactLab for Applied Social Science Research, University of North Texas, Denton, TX 76203, USA; evaperezzepeda@my.unthsc.edu

**Keywords:** food insecurity, international graduate students, higher education, ethnographic research, cultural food access, student well-being, campus food pantry, qualitative methods, basic needs insecurity

## Abstract

Background/Objectives: International students pursuing higher education in the United States face unique challenges that increase their risk of food insecurity, including limited financial resources, employment restrictions, and cultural barriers. While food insecurity among domestic students has been widely studied, limited research focuses on the lived experiences of international graduate students. This study explores the challenges, perceptions, and coping strategies related to food insecurity among international graduate students at a large public university in North Texas. Methods: This qualitative, ethnographic study involved 20 semi-structured interviews with international graduate students who were clients of the university’s food pantry. Participants were recruited using purposive convenience sampling. Interviews focused on students’ experiences with food access, financial constraints, campus resources, and cultural food preferences. Data were analyzed using thematic coding in MAXQDA. Two standardized food insecurity measures—the USDA and FAO scales—were also administered and analyzed using SPSS. Results: Findings revealed that 85% of participants experienced limited access to nutritious and culturally appropriate foods, with 70% reporting hunger due to financial constraints. Themes included lack of cooking skills, limited campus food options, difficulty accessing familiar groceries, and limited job opportunities. Students expressed that food insecurity significantly impacted their physical health, mental well-being, and social lives, though many continued to prioritize academics over personal nourishment. Conclusions: Food insecurity among international graduate students is multifaceted, shaped by financial, cultural, and institutional barriers. Addressing this issue requires culturally sensitive interventions, improved access to diverse food options, tailored student support services, and institutional efforts to better understand and meet the needs of international students.

## 1. Introduction

### 1.1. International Students in Higher Education

In the past decade, the number of students traveling abroad for higher education has increased [1,2,3,4,5,6], especially in English-speaking countries like Australia, Canada, the UK, and the United States [5,7]. International students have expectations that programs in these countries are of better quality or are unique and different from programs in their home country [2]. In the United States, international enrollment has steadily increased since the 1950s, especially in the last two years [7]. In 2023, there were over 1.5 million international students enrolled, a 10.4% increase from 2022 [8]. Texas is a popular destination for international students, ranking third among U.S. states that hosted the largest percentage of international student visas in 2023 [8,9].

While international students seek to enroll at all levels of higher education [7,10], the majority seek graduate degrees. Master’s degrees (45%) and doctoral degrees (21%) account for 66% of the higher education degrees sought by international students [8]. The *Snapshot on International Student Enrollment* revealed an 18% increase in graduate student enrollments in Fall 2022 and another 7% increase in Fall 2023 [11,12].

In 2023, international students from over 238 countries studied in the United States. Asia, overwhelmingly China and India, accounted for over 70% of these students [8,9]. While China saw a slight increase of 1.9% (+6169 students) in 2023, India increased its number of students studying in the U.S. by 27.1% (+80,469 students) [8].

There are several reasons why U.S. institutions want to host more international students. The U.S. higher education system is facing a sharp decline in domestic students, with nearly a 10% decrease since 2010 and a projected 15% decrease by 2025 [7]. A Fall 2023 survey revealed that 92% of institutions want to increase international enrollment, and 85% have financially supported recruiting international students [7]. International students serve as a major source of revenue for universities as well as their host countries, contributing over USD 43.8 billion to the U.S. economy and supporting 378,175 jobs in the 2023–2024 academic year alone, a 10 percent increase from the previous year [13]. Beyond an economic impact, it is thought that these students enrich universities and states with global perspectives and cultural diversity [1,7,14,15].

Despite the growing efforts of recruiting international students and their significant contributions to their host institutions and countries, many students face financial challenges due to high tuition costs, limited funding opportunities, employment restrictions, and housing and cost-of-living expenses [2,5,10,15,16]. According to Calder et al. [15], some international students in North American universities provide misleading information about their financial resources in order to meet the required minimum bank account balance (which can be USD 10,000 or more, depending on the university) required to get a student visa, and many of these arrive without a clear understanding of the true cost of living. They may not have access to relevant or adequate information about living expenses, leading to financial difficulties [15]. In the U.S., although on-campus jobs are available for international students, they are very difficult to obtain. The rising costs of tuition, housing, and living expenses, coupled with limited financial aid options, suggest that many international students may struggle to afford basic necessities, like food and housing [17,18].

### 1.2. Food Insecurity Among College Students

Food insecurity is defined as the “limited or uncertain availability of nutritionally adequate and safe foods or limited or uncertain ability to acquire acceptable foods in socially acceptable ways” [19]. Food insecurity prevalence among the general U.S. population increased from 2022 (12.8%) to 2023 (13.5%), reaching its highest level since 2014. Texas is one of seven states with food insecurity above the U.S. average [19].

Research studies indicate that college students have higher prevalence rates of food insecurity than the rest of the population [4,20,21,22], making this a prevalent issue on college campuses across the U.S. [23,24,25]. A 2020 scoping review of food insecurity among U.S. college students found that food insecurity rates ranged from 10% to 75%, estimating that food insecurity is experienced by 41% of college students [21]. Federal data from the 2019–20 National Postsecondary Student Aid Study survey revealed that 22.6% of undergraduates reported low or very low food security, and another 11.9% reported marginal food security, totaling 34.5% experiencing some degree of food insecurity. For graduate students, 12.2% reported low or very low food security, and 8.6% reported marginal food security, totaling 20.8% experiencing some degree of food insecurity. This translates to more than 4 million higher education students (3.9 million undergraduate and more than 400,000 graduate students) experiencing food insecurity, with an additional 2.3 million experiencing marginal food security [26]. These statistics highlight the urgent need to address this critical challenge within higher education.

Research focusing on food insecurity among college students has increased rapidly in recent years [21,27], revealing food insecurity trends among specific at-risk sub-populations of students such as marginalized racial groups [22,27,28], those with disabilities [28], undocumented immigrants [29], and LGBTQ+ students [25]. Hammad and Leung [27] researched the unique experiences of graduate and post-doctoral students at Harvard University. Risk factors for food insecurity included being Asian, belonging to a racial or ethnic minority group, housing instability, and lack of transportation. Graduate students with financial struggles and heavy workloads are particularly vulnerable to food insecurity [27]. A similar trend can be seen with international students.

### 1.3. Food Insecurity Among International Students

Being an international student is a risk factor for food insecurity. Several published studies reveal that international students have a higher prevalence of food insecurity than their domestic counterparts, including out-of-state students [10,30,31,32]. A 2021 systematic review found that international students were two to three times more likely to experience food insecurity and more likely to use food banks than domestic students [4]. A 2022 study at a Southeastern university found that one in four international students were food insecure, and another one in four were marginally food insecure [10]. A 2024 cross-sectional study conducted at a large Texas university further confirmed this trend, with 32.4% of their international students reporting being food insecure [16]. A qualitative study from four universities across the US found that international students faced significant dietary challenges, with health consequences including weight gain, abdominal discomfort, and health issues like acid reflux, ulcers, and acne [33]. The majority resorted to taking dietary supplements after moving to the United States.

The association between food insecurity and poorer health outcomes, mental health, and academic performance and success is well-documented among college students in general [4,22,34,35,36,37,38,39,40,41]. In addition to the increased risk of being food insecure, international students have additional compounding factors that impact their well-being and success. They are more likely to experience a range of psychological challenges, including anxiety, loneliness, and depression [2]. Additionally, the challenges of adapting to a new culture, foods, language, and academic system can lead to significant stress and anxiety [1,3,42,43]. Cultural shock, discrimination, and a lack of social support can further isolate and marginalize international students, contributing to their overall psychological well-being. These factors can have a profound impact on students’ academic performance, leading to lower grades [44,45], hindering educational attainment outcomes [41], and health consequences [33,41,46].

The existing literature highlights the critical nature of food insecurity among international students and emphasizes the need to further investigate this population and generate culturally and contextually relevant strategies [15,30,47,48]. This qualitative, ethnographic study aims to explore the experiences of food insecurity among international graduate students at a large public university in North Texas, ranked among the top 20 SEVP-certified schools for F−1 students in 2023 [8]. According to the 2023 National College Health Assessment, international graduate students have one of the highest rates of food insecurity at the university [49]. By understanding the impact food insecurity has on their overall well-being, this research seeks to recommend culturally sensitive solutions to support international graduate students.

### 1.4. Research Questions

1. What are the experiences of food insecurity among international students seeking a graduate degree at a large university in Texas?
2. What is the perception of food insecurity among international students?
3. What are some of the challenges that contribute to food insecurity among international students?
4. What solutions would international students like to see?

## 2. Setting and Methods

The research setting was a large public research university of >40,000 students in Texas. At the time of study (spring 2024), the university hosted 8276 international students, most of them seeking graduate degrees; 7000 (84%) were residents of India, Nepal, Bangladesh, or Pakistan. In fall 2023, South Asian graduate students comprised 85% of the population of users of the university’s food pantry. The researchers for this project are (1) a medical anthropologist who has researched college food insecurity for ten years, (2) a medical anthropologist who led an Anthropology in Public Health class with undergraduate/graduate students to conduct this research in 2024, and (3) a master’s student in applied anthropology and public health.

An applied ethnographic and qualitative approach was used for this study to capture international students’ firsthand accounts of food insecurity and understand the meanings they attach to it, as well as their strategies for navigating hunger while pursuing their degrees. Ethnography offers a flexible approach to research that prioritizes the voices of participants, allowing them to articulate their experiences and perspectives in their own words. Researchers use ethnography to uncover the nuances of human behavior within its broader cultural and social context [50,51,52,53]. Researchers use applied ethnography to identify key issues in a community and apply those results to bring about beneficial change [52].

The research population for this project focused on current South Asian international graduate students who were clients of the campus food pantry and who self-identified as food insecure. Purposive convenience sampling was used to recruit participants. The associate director for the food pantry emailed a recruitment flyer to all international graduate students who were previous or current clients of the pantry. Interested students contacted a lead researcher who asked them screening questions to confirm their qualification for the study.

Data collection consisted of 20 semi-structured interviews lasting an average of 45 min. The interview questions were designed to understand and contextualize the participants’ experiences of food insecurity as international students. Interview topics focused on perceptions of food insecurity, challenges, impacts, and coping strategies. Other topics included how students decided on this university, their experiences when they first arrived, how they learned about the university’s food pantry, their experiences with it, and general questions about how the university could better support international students like themselves (see Appendix A).

Interviews were conducted in person or over Zoom. All participants gave informed consent, obtained in accordance with IRB approval, which outlined the study’s purpose, benefits, and potential risks associated with participation. A pilot interview was conducted prior to data collection to ensure appropriateness and sensitivity were achieved. Interviewers were trained to follow the interview guide consistently and to probe for additional information when necessary.

The lead researcher used MAXQDA to code and analyze the data. Initial themes were derived directly from the research questions, while additional subthemes emerged from the data analysis to provide a more detailed understanding of participants’ responses [52]. To ensure privacy, all collected data remained confidential using coded identifiers.

We also assessed food insecurity using two accepted quantitative measures of food insecurity. One scale was by the US Department of Agriculture (also used by the National Collegiate Health Assessment on the campus). The second scale was developed by the United Nations Food and Agriculture Organization (FAO), which has cross-cultural validation. SPSS (Version 29) was used to analyze the results of these.

Participants in this study received three USD 10 meal vouchers for campus dining services provided by the Division of Student Affairs.

## 3. Results

### 3.1. Demographics

Our sample consisted of 20 total participants (see Table 1): 13 males and 7 female participants. Their ages ranged from 22 to 37 years. Fifteen students were originally from Southern India, two students were from Sri Lanka, one from Pakistan, one from Nepal, and one student did not disclose their country of origin. We recruited students pursuing postgraduate degrees (master’s or PhD). Twelve participants were in their first year, while eight were in their second year.

### 3.2. Food Insecurity Survey Results

Based on the United Nations questions, a majority of our respondents reported being worried about running out of food within the past 30 days (70%), eating less than they thought they should (60%), or skipping meals (63%) (see Table 2). Many reported eating only a few kinds of food (85%) and struggled to eat healthily (85%). Four of twenty (20%) said they went a whole day without eating.

The USDA survey revealed similar trends (see Table 3). Sixty percent of participants reported food not lasting as long as they thought it would last, and did not have money to get more in the last 30 days. Eighty percent reported not being able to afford to eat balanced meals. Additionally, 70% experienced hunger but could not afford to eat, 75% reduced meal sizes or skipped meals, and 80% consumed less food than they believed was necessary due to financial limitations.

### 3.3. Perceptions of Food Insecurity

Interviewers asked participants to explain the meaning of food insecurity in their own words to assess unique perceptions based on the cultural or contextual circumstances of this population. For the sample, food insecurity was a complex web of challenges—a situation where students must make tough choices about what to eat because of financial constraints or limited availability of healthy options when compared to access to food in their home country. For international students, the term food insecurity had multiple dimensions. These included having limited or uncertain access to nutritious and culturally appropriate food, concerns over the quality of their food, uncertainty about meals and meal access, limited choices, financial limitations, and personal struggles.

One student defined food insecurity as:

“*Food insecurity, for me, if I’m not able to avail or provide myself with sufficient nutrients for the day, due to lack of resources, or some constraints, … If I am not adequately provisioning myself, nurturing myself, that is food insecurity.*”

For many international students, food insecurity meant not having enough nutrients or compromising nutrition. They noted the abundance of processed, preserved, and pre-packaged foods available in American grocery stores in stark contrast to the fresh fruits, vegetables, and meats readily available and affordable in their home countries. They found themselves choosing cheaper food options, even when they were unhealthy. A student mentioned, “*I had never seen potatoes being canned. I wasn’t even aware you could do such a thing. In India we’re used to a lot of fresh produce. We never preserve.*”

### 3.4. Limited Food Choices

Limited access to culturally appropriate food was a major concern for many students, especially for those with vegetarian, vegan, or religious dietary restrictions. As most of these students were new to the area around campus, they were not familiar with local ingredients or culturally appropriate substitutes for their usual foods. This was challenging because unlike back home, where familiar shops and ingredients are readily available, international students struggle to navigate grocery stores and find affordable, healthy options. This was especially true in the first one to six months of their arrival to the U.S. One student mentioned, “*For the first six months, I was really starving for like one or two days because I couldn’t find the proper food. I couldn’t find a way or find the place to find the food.*”

Even though the university’s dining halls offer plant-based options, students report mixed accessibility to these foods. Some students acknowledged these options in a positive light, stating how helpful these options are, while other students expressed that there are times when the choices are very scarce, and they would have to explore off-campus to find options.

Furthermore, students mentioned that they preferred to buy their groceries from Indian grocery stores. However, some international students mentioned how “unpredictable” food availability is at times, even at these stores. For example, one student mentioned an incident of stocking issues that limited their resources for familiar foods. Similar issues happen with the campus food pantry since what is available at the pantry can vary with every visit.

### 3.5. Financial Limitations

The interviews highlighted the severe economic hardships faced by international students. As newly arrived students, they have limited financial resources and several essential expenses in the first few months. Students said they were unaware of the high cost of health insurance payments and the general high cost of living in the United States, making it difficult to afford food. A student shared, “*I pay $900 monthly for my apartment and $600 for health insurance, so I am left with something small to buy food. I cannot allocate more money for food.*”

International students also mentioned how they came to the U.S. with educational loans, but that money only covers part of their expenses. Student testimonies share instances of starving themselves for days waiting for their next deposit or stretching their food budgets.

“*My journey as an international student at the university …[for] the period when I was unemployed—so I found myself struggling to make ends meet and especially when it came to putting food on the table. Because as a new international student, I had many other things to buy with limited financial resources. I was shedding every dollar to cover those essential expenses, including groceries. So, there were times when I had to make difficult choices between paying bills and buying food. One time I had exhausted the last of my groceries and felt a sense of desperation wash over me. So, in that moment I realized the reality of food insecurity. Despite my best approach to budgeting, like expenses, I found myself facing hunger and anxiety about where my next meal would come from. So, it was a humbling experience.*”

While the university promotes on-campus work opportunities for incoming students, many found securing a job very challenging. Seven international students expressed disappointment in their inability to find on-campus employment despite initial expectations. Not securing an on-campus job contributed to their financial insecurity. Students mentioned the need to extend their loan amounts for survival. This often led to them sacrificing food intake at worst or food quality at best, resorting to fast food or what they considered to be less nutritious but cheaper processed options. However, even for those who secured on-campus jobs, the pay was not what they expected, and the lack of income during academic breaks was a challenge for them.

“*Most times it is hard for me. I only eat well within the first week that I am paid. And in [the] Physics department, we have funding for only 9 months and so for the other months that school is on vacation, I really find it difficult to eat because I am not being paid. With my status, I cannot work off campus to gain extra income which is a big problem.*”

### 3.6. Balancing New Responsibilities

Learning how to balance academic, personal, and financial responsibilities can be a significant challenge for international students. Many reported prioritizing academics and needs like rent and utilities over healthy food, leading to compromised dietary choices. Juggling a demanding academic schedule, part-time jobs, and other responsibilities leaves students with little time to cook healthy meals. One student mentioned:

“*It’s because many priorities…and we [my roommates] sacrifice food first. We just think about the living, staying on the course. And it’s pretty normal, I would say. You know, I have been through this in two apartments, and we sacrifice food first thing. When it comes to insufficient funds, they prioritize their courses, their sleep, they stay calm, they don’t do anything, they don’t go anywhere, they just spend their days just without eating anything much.*”

Students, particularly those in their first year, may not have the necessary cooking skills or experience to prepare nutritious meals on their own. Many international students, particularly male participants, mentioned that when they first moved to the U.S., they did not have the necessary cooking skills or experience to prepare their meals and had to learn how to cook. Back home, they relied on their parents to cook. This created an adjustment period for international students to figure out how they were going to feed themselves sustainably. As one participant shared, if you do not know how to cook, you will end up eating something unhealthy. Many students resort to quick and convenient, often less healthy, food options to save time and money. A student shared their experiences when it came to cooking:

“*I think the first basic thing that we, as students, if you don’t know how to cook, that’s where you’ll start having the insecurity. If you cannot cook, if you don’t know how to cook, you will probably end up eating something unhealthy. So, the amount of effort that I put into cooking, the output wasn’t right. So, I had to throw it. So that’s where I get fed up with even cooking and I’ll be like I’m done with this cooking. So, food is not even a priority for students sometimes I’d say*.”

“*I think it’s all because when I wanted to eat something, I didn’t know how to cook it. I ended up eating something else to manage that hunger, but that hunger didn’t end. And then I ended up started eating sugars (laughs).*”

### 3.7. Campus Challenges

Most of our participants did not have a campus meal plan, so if they needed to eat food from campus, they had to spend money. The university has several dining halls and eateries, but food prices vary. Most participants took classes on a satellite campus, where the food options are more limited and more expensive than the main campus.

The main campus provides a food pantry accessible to all students, which served as a supplement for the international students in our study. However, our sample identified limitations of the food pantry. Indian students, for example, emphasized the importance of certain types of wheat and rice, noting difficulties in finding these staples at the campus food pantry. Additionally, the pantry hours did not always work with their demanding schedules, and they were limited in the number of items they could take. This discouraged some of them from using the pantry and incentivized them to utilize other sources for groceries. First-year students used the pantry more often than those in their second year.

The lack of personal vehicles and the lengthy commute to grocery stores using public transportation made regular grocery shopping difficult and time-consuming. An Indian student described the exhausting process of taking several buses to stock up on groceries, often resulting in spoiled food when they reached home. Another student discussed postponing grocery shopping if they had difficulties scheduling ride-share transportation.

“*Because we don’t know our way when we first start here, and we use public transport, we used to walk to Union, take the bus to [the shopping area], get stuff, and then get the bus back and walk all the way home. So, you know, we don’t want to do it very often because it’s a tremendous process. And we go on the bus and can see the food [rotting].*”

### 3.8. Impacts on Health and Well-Being

Food insecurity among higher education students can have significant impacts on their physical and mental well-being [34,36]. The international students in our sample mentioned that the challenges in accessing culturally appropriate and nutritious foods had impacts on their physical and mental health and their overall well-being.

Physically, international students reported experiencing issues associated with inadequate nutrient intake, such as significant weight loss. Several students reported weight loss, but one student emphasized the drastic change in the first two months of being in the U.S.:

“*I was around 82 kilos when I came down in August. Within two months of me not knowing how to cook and depending on the groceries or my friend’s cooking and their experiments, I lost 15 kilos. Within the two months*.”

In addition to weight loss, participants identified many other physical health problems, including fatigue, weakness, and digestive issues. Skipping meals often led students to develop headaches, dizziness, and sometimes faint. This caused difficulty concentrating in class and being less physically active than they used to be. Moreover, female students identified disruption in menstrual cycles, leading to pain and discomfort, which they attributed to inadequate nutrition. One student stated that during her menstrual cycle, she would also experience a “*lack of strength and stamina*” that would last one or two days.

The psychological impact of food insecurity is significant. Although participants try to keep their mental health strong, combined stressors of being away from family, dealing with family issues, cultural adjustment, the stress of academic demands, and navigating economic challenges in the U.S. take a significant emotional toll. On the one hand, the constant worry about where their next meal will come from and the stigma associated with food insecurity led to feelings of nervousness and stress. On the other hand, the amount of stress that international students deal with also disrupts healthy eating patterns. One student mentioned how a family emergency caused them stress and often made them lose their appetite. When they came back from visiting their home country, they did not eat properly for almost a month. Another student expressed a similar sentiment:

“*…but also, the stress. Any family stress or parents…Any stress that goes on, and for students like us if we are taking stressful eating and everything it probably impacts your health. Mentally, physically. And obviously you don’t even feel like eating if you are so [stressed]. That’s something.*”

Finally, the financial constraints associated with food insecurity limit social and physical activity. Participants reported that they avoid going out in order to manage financial strain.

### 3.9. Coping with Food Insecurity

International students mitigate food insecurity in a variety of ways. Those who are better equipped to cook find it more cost-effective to cook and eat at home than dine out, especially if they have food restrictions (e.g.,Halal). One student mentioned:

“*So basically, finding time is really hard as a younger person and who is managing studies and part time work, and then coming home doing things, organizing everything is very hard. You get a very limited amount of time. But whenever I get time, like a very few times, such as whenever there is a break of five days, or there is a one weekend holiday, I would plan my meals, and I would try to create a weekly plan based on all the budget I have. And I try to buy nutritional ingredients, such as greens, legumes, fruits and vegetables. I try to use the coupons to maximize my savings are things that I would follow.*”

However, for students who are not as comfortable with cooking or those who want to allocate more time to their studies, a common strategy was to look for cheaper food options that can be prepared quickly. This helps when they are in a rush to class in the morning, or when they are busy throughout the day. Often these foods include snacks like fruits, nuts, peanut butter, bread, bananas, fries, or instant meals, which are cheaper than campus food option. One student shared:

“*Like in the grocery stores, probably I’ll get these instant meals. Like, at that moment itself I can just, you know, stir it, boil it, eat it. It’s just within five minutes. It saves your time, it does also mean that you’ve eaten, and you’ve already followed your schedule. I mean, you have to buy that as well.*”

Other coping strategies include attending university free food events and having liquids like warm water, tea, or milk to subside hunger.

International students prefer to get their groceries from Indian stores, as they are more likely to meet their cultural needs and are slightly less expensive than mainstream grocery stores. However, at the time of the study, most of these stores were more than 30 miles away. Carpooling and buying enough food for two weeks were common. If their schedules became too busy, they would go to closer ethnic stores, but only in “emergency situations.”

Another way in which they met cultural needs was by seeking things that felt familiar. For example, the popularity of food trucks and food stands in India was mentioned by students. Around campus, a few food trucks with South Asian food have opened during the past year, providing a variety of dishes from fried foods to curries and rotis. One student mentioned how these food trucks offer a convenient way to satisfy food cravings, especially for midnight snacks or when grocery shopping is not feasible. Essentially, food trucks are filling a gap in the market by offering a taste of Indian street food culture in a convenient and accessible format.

“*This semester there are a lot of Indian foods, like the new places with Indian food, but earlier, there was nothing*.”

## 4. Discussion

The increasing global trend of international student mobility has brought significant benefits to higher education institutions and host countries [1,7,14,15]. However, it has also highlighted the challenges faced by these students, particularly in terms of food and financial stability [4,10,16,30,31,32]. This study researched the experiences of food insecurity among international graduate students at a large public university in North Texas. Specifically, the study population included international clients of the university’s food pantry.

Similar to domestic students, factors like financial constraints and academic and personal demands contributed to food insecurity among our sample. For example, respondents faced challenges in maintaining a healthy diet due to financial constraints. The high cost of living in the United States, coupled with limited financial aid options, made it difficult for many students to afford nutritious food. While the additional income was helpful for students who were able to obtain an on-campus job, it is not substantial enough to fully cover living expenses. One student noted that their on-campus job allowed them to qualify for in-state tuition, which decreased some of the financial burden, but argued that the hourly wage was not high enough to offset their financial burden.

It has been widely documented that students who are struggling to meet their basic needs find it difficult to focus on their studies, leading to lower grades and increased risk of academic failure [38,39,54]. While some international students in our sample mentioned instances of not being able to concentrate during class because of hunger, our sample did not identify academics as an area that was impacted by food insecurity. They came to the United States to advance their education, and they made this their main priority at the expense of adequate and nutritious food.

International students have unique experiences that contribute to their food insecurity. Within our sample, international students experienced trouble accessing culturally appropriate food, navigating unfamiliar grocery stores, and having limited cooking skills as newly arrived students. Foods familiar to one’s home culture are a source of nostalgia and comfort [42], so having access to culturally appropriate food is essential for these students especially when they have feelings of homesickness and isolation. One student shared their experience with homesickness:

“*Yeah. I felt homesick. You know, obviously. I mean in the initial three months I was so overexcited, I was so happy about being here, I was part of each and every club, rec sport, I would say every event. But then with the sudden shift of the apartment and me starting the budgeting and everything my well-being, my state of mind, my living experiences changed. So, I did feel like—this is not something I expected and I feel like I am struggling right now. And I felt homesick*.”

The findings of this study have significant implications for higher education institutions. Improvements targeting the experiences of international students should be built upon the locally identified challenges and the significance of food insecurity. International students in particular may have a unique array of needs, from realistic financial counseling that understands the pressures that international students face, food pantries with diverse food options, time management skills, guides to ethnic groceries and local transportation in the region, and cooking workshops. All institutions recruiting international students should consider the cultural and dietary needs of international students when designing food programs and services, in order to ensure diverse, culturally appropriate, and affordable meal options [55]. For example, dining services staff could meet with international student representatives and cultural advisors to regularly review and update menu offerings.

For universities recruiting international students, developing a comprehensive orientation program and support services that address the specific needs of international students is essential. Tailored, in-person orientation programs should cover topics such as cultural adjustment, academic expectations, and practical aspects like financial planning, regional transportation, and food access. Workshops could be led by current international students who provide peer insights into navigating daily life, including grocery availability, cost, and meal preparation. Mentorship programs could also be implemented, as they have proven to be effective in the past [56,57,58]. As we noted above, participants who were on their second year at the university explained that they identified as food insecure during the first few months in the United States, but after a few months they learned to “manage it.” This data supports Ibiyemi et al.’s [16] observation that “duration of stay in the U.S. [is] a predictor of food insecurity status” and emphasizes the importance of considering mentorship programs. International students could be paired with experienced mentors from the same host country who have been in the U.S. for 2–3 years, who can provide guidance, support, and advice on navigating academic, financial, and social challenges. Existing mentorship programs could be strengthened by focusing on issues and solutions around food insecurity, including food access, budgeting for food, and the availability of campus and community resources. Research shows that discussing food insecurity helps students to understand that they are not alone in this situation [23].

There is an opportunity to provide financial support and empowerment as well. By offering workshops on budgeting, money management, and understanding U.S. financial systems, universities can help students make informed financial decisions that work best for them. Advocating for increased financial aid opportunities, including scholarships, grants, and improved on-campus work opportunities, is something that would help alleviate financial burdens for international students. When asked how the university can help alleviate their problems, they mentioned the following:

“*Yeah, I mean, to eliminate my problems here they can provide me a job here! So that I can have some money, so that the groceries and rent I can pay groceries and rent here. So that it would be helpful for me… but it’s very competitive because there’s so many students.*”

Finally, research shows that university food pantries provide some support, but limited resources and accessibility constraints are insufficient to address the widespread need. Additional support should include increasing the diversity of food items (including fresh produce and cultural relevant foods), expanding hours, and ensuring location accessibility, particularly for large campuses.

## 5. Limitations and Future Research Directions

Although two established food insecurity scales were used, our research suggests that items within these scales may not be valid for all populations. Others have pointed out the problematic nature of how college students interpret common food insecurity scale items like “household” and even “money,” and more research needs to investigate how college students’ experiences with food insecurity may differ from the general population [27,59].

Future work should critically examine the validity and reliability of these scales when applied to international students, as our ethnographic data suggest that the constructs used would not accurately measure the outcomes for which they are designed. Some of this is in the wording of individual items. For example, as we detail above, what is “*healthy and nutritious*,” or what is “*a balanced meal*” both have culturally constructed meanings, and when an international student used to a diverse, fresh, and culturally or religiously sanctioned diet is suddenly faced with narrower and foreign food options, they are unlikely to see this change as “healthy and nutritious.” For South Asian students in particular, used to the easy availability of a wide variety of fresh fruits, vegetables, legumes, and whole grains, adjusting to an American diet replete with processed or canned foods could be difficult. As well, particularly the first semester, many international students must learn how to juggle new transportation realities needed to access healthier foods, new time management skills, and even (particularly men) how to cook for themselves for the first time. Therefore (as our participants noted above), “*skipping a meal,*” “*having an apartment run out of food*,” or eating “*only a few kinds of foods*” are situational realities that international students find themselves in that can become resolved after 1–2 semesters. Because our research shows such differences in reliability between how international students may interpret food insecurity scale items, it is imperative that food insecurity measures be constructed that are validated for this particular population. This would allow research to more accurately comprehend the place of food insecurity among international students studying abroad.

Finally, further research is needed to explore the long-term consequences of food insecurity on international students’ academic performance, mental health, and overall well-being. Quantitative data should be collected to measure the impact on academic performance, such as graduation rates or grade point average. 

## 6. Conclusions

Food insecurity among college students has increasingly become a topic of concern. By understanding the multifaceted challenges faced by international graduate students, we can work towards developing comprehensive solutions to address food insecurity and promote the overall well-being of this vulnerable population.

## Figures and Tables

**Table 1:** Demographics of the sample (N = 20).

Demographics		Sample Frequency	Sample %
Gender	Male	13	65%
	Female	7	35%
Degree	Master’s	18	90%
	PhD	2	10%
Year	1	12	60%
	2	8	40%
Country of origin	India	15	75%
	Nepal	1	5%
	Pakistan	1	5%
	Sri Lanka	2	10%
	Unknown	1	5%

**Table 2:** UNFAO Food Insecurity Scale results (N = 20).

UNFAO Food Insecurity Scale	Sample Frequency	Sample %
Were worried they would not have enough to eat	14	70%
Were unable to eat healthy and nutritious food	17	85%
Ate only a few kinds of foods	17	85%
Ate less than they thought they should	12	60%
Had to skip a meal	14	63%
Apartment/house ran out of food	10	50%
Were hungry, but did not eat	14	70%
Went a whole day without eating	4	20%

**Table 3:** USDA Food Insecurity Scale results (N = 20).

USDA Food Insecurity Scale		Frequency	Sample %
The food that I bought just didn’t last as long as I thought it would last, and I didn’t have money to get more (30 days).	Never	8	40%
	Sometimes	7	35%
	Frequently	5	25%
I couldn’t afford to eat balanced meals	Never	4	20
	Sometimes	8	40
	Frequently	8	40
Did you ever cut the size of your meals or skip meals because there wasn’t enough money for food? (30 days)	No	5	25
	Yes	15	75
Did you ever eat less than you felt you should because there wasn’t enough money for food? (30 days)	No	4	20
	Yes	16	80
Were you ever hungry but didn’t eat because there wasn’t enough money for food (30 days)	No	6	30
	Yes	14	70

## Data Availability

Due to the sensitive nature of this qualitative study, which involved collecting detailed personal narratives and explanations from participants, and in accordance with the approved IRB protocol, the dataset will not be made publicly available.

## Appendix A

**Sample Semi-Structured Interview Questions**

1. We’ve heard that (the University) receives students through recruiters/“consultants” in India/ South Asia? Did you use one? What is that experience like? What did they tell you about (the University) in the beginning?
2. (The University) tells us that they advise international students on how much to budget monthly for food and apartments. Did you receive this advice?
3. (If yes). How would you compare your actual experience with budgeting for food and rent with what they told you/advised you?
4. How much do you yourself budget for food in a month? (groceries, eating out, meal plans). Is it enough to get you through the month? Do you have a meal-plan?
5. Is there anything you wish you knew before you got here?
6. How does the reality of graduate school at (the University) compare to what you thought it would be?
7. How (and when) did you first hear about the food pantry? What did you hear?
8. What does “food insecurity” mean to you?
9. Do you feel like “hunger” or being “food insecure” applies to your own situation? Can you explain? (Tell us about food insecurity as a college student?)
  (10b) (IF NO) So then what, for you, is the purpose of the food pantry? (SKIP TO Q19).
10. (IF YES) What are some of the challenges that make it hard to be food secure?
11. (IF YES) When did you realize that food, budgets, and hunger were going to be problems?
12. (IF YES) Are there any particular times that you feel MORE food insecure than other times?
13. (If YES) Do you have strategies to cope? Other than “not eating?”
14. (IF YES) Has being food insecure impacted your college experience? How? How about your studies?
15. (IF YES) Had you ever experienced food insecurity before attending (the University)?
16. (IF YES) Do you feel like this food insecurity has impacted your physical health?
17. (IF YES) How about mental health?
18. Do you feel like your food/ hunger situation matches other international students? How?
19. One of the questions in the scale we started off with asked if you could afford to eat “balanced” meals? What is a “balanced” meal to you? How about “nutritious” foods?
20. What kinds of food do you eat here? Is that different than what you grew up with? Do you have food restrictions?
21. Are there some foods that you need, but don’t have access to? (and why?)
22. How do you typically get food? What are your main sources of food?
23. About what % of your groceries come from the University food pantry, what % come from an American grocery store like Walmart/ Kroger/Winco, and what % come from a South Asian grocery store you visit? Somewhere else?
24. When you get foods, what food is typically the priority for you?
25. So I know you did explain that you have gone to the food pantry before. Can you tell me about that experience?
26. So how many times in a typical month do you go?
27. Are there any barriers you have to using the pantry? (probe)
28. Are there items you would like to see more of there?
29. Is the quality OK? (explain)
30. How could (the University) better support international students?
31. How could (the University) help alleviate some of your current problems?
32. Is there anything different that (the University) should tell international students before they get here? Would that have made a difference?
